# Role of Adaptor Protein Myeloid Differentiation 88 (MyD88) in Post-Subarachnoid Hemorrhage Inflammation: A Systematic Review

**DOI:** 10.3390/ijms22084185

**Published:** 2021-04-18

**Authors:** Hammad Ahmed, Mahtab Ahmad Khan, Ulf Dietrich Kahlert, Mika Niemelä, Daniel Hänggi, Shafqat Rasul Chaudhry, Sajjad Muhammad

**Affiliations:** 1 Department of Basic Medical Sciences, Imran Idrees College of Pharmacy, University of the Punjab, Sialkot 51310, Pakistan; drhammadahmad@live.com; 2 Department of Pharmacology, Faculty of Pharmacy, University of Central Punjab (UCP), Lahore 54000, Pakistan; mahtab.ahmad@ucp.edu.pk; 3 Department of Neurosurgery, Medical Faculty, Heinrich-Heine University of Düsseldorf, 40225 Düsseldorf, Germany; ulf.kahlert@med.uni-duesseldorf.de (U.D.K.); daniel.haenggi@med.uni-duesseldorf.de (D.H.); 4 Department of Neurosurgery, University of Helsinki and University Hospital, FI-00029 HUS Helsinki, Finland; mika.niemela@hus.fi; 5 Department of Basic Medical Sciences, Shifa College of Pharmaceutical Sciences, Shifa Tameer-e-Millat University, Islamabad 44000, Pakistan; shafqatrasul@yahoo.com

**Keywords:** stroke, pattern recognition receptors, aneurysms, neuro-inflammation, post-SAH complications

## Abstract

Myeloid differentiation 88 (MyD88) is a well-established inflammatory adaptor protein. It is one of the essential downstream proteins of the toll-like receptor 4 (TLR4) signaling pathway. TLRs are pattern recognition receptors that are usually activated by the damage-associated molecular pattern molecules (DAMPs). Sterile inflammation is triggered by the endogenous DAMPs released in response to global cerebral ischemia and from extravasated blood after subarachnoid hemorrhage (SAH). In this review, we highlight the importance of the neuroinflammatory role of the MyD88 in the SAH. We also explore a few possible pharmacological agents that can be used to decrease SAH-associated neuroinflammation by modulating the MyD88 dependent functions. Pharmacological agents such as flavonoids, melatonin, fluoxetine, pentoxifylline and progesterone have been investigated experimentally to reduce the SAH-associated inflammation. Inhibition of the MyD88 not only reduces the expression of pro-inflammatory cytokines, but also potentially inhibits other processes that can augment the SAH associated inflammation. Further investigations are required to translate these findings in the clinical setting.

## 1. Introduction

Subarachnoid hemorrhage (SAH) is a subtype of hemorrhagic strokes and accounts for only 5% of all strokes [1]. However, the morbidity and mortality inflicted by SAH is a nightmare in this era of modern medicine as reflected by the fact that 20% of deaths occur before hospitalization and half of the patients by getting medical care die within a month [2,3]. The condition of the survivors is also not very good and almost one-third are lifelong dependent and many of the survivors with good clinical outcomes still have cognitive disorders [2]. SAHs are of concern compared to ischemic strokes as they tend to affect a younger population including those of working age group [1]. SAH accounts for almost 27% of stroke-related potential years of life lost by an individual before the age of 65 years [4]. The majority of SAH cases are due to the rupture of abnormally dilated, ballooning and weakened intracranial blood vessels at the arterial bifurcations; these abnormal rupture-prone arterial dilatations are referred to as intracranial aneurysms (ICAs) [5]. Immediately, after the rupture of ICAs, the subarachnoid space is flooded with extravasated blood with an elevation in intracranial pressure, which compromises the normal circulation of cerebrospinal fluid (CSF), ultimately leading to transient global cerebral ischemia [1,6]. The toxic effects of the blood and its degradation products further add to the injury of the brain [1,6]. There is a considerable body of evidence suggesting that during this insult, several damage-associated molecular patterns (DAMPs) are liberated from various cellular compartments, which have the capacity to upregulate inflammation after ligation of their cognizant pattern recognition receptors (PRR) [7,8,9,10,11]. Over the past few years, the concept of early brain injury and delayed brain injury has evolved to consider the events triggered immediately after the sentinel bleed up to 72 h and over 3–14 days or more, respectively [1,6,12,13]. Sterile inflammation mediates both of these injury phases and also contributes to various post-SAH complications such as cerebral vasospasm (CVS), seizures, delayed cerebral ischemia (DCI), cortical spreading depolarization (CSD), chronic hydrocephalus and infections, etc. that ultimately affect the clinical outcome of patients [9,10,14]. Therefore, local and systemic inflammation after SAH has been the target of current, intensive investigations to uncover the underlying complex mechanisms and highlight them as important drug targets.

The innate immune response relies on several germ line-encoded PRRs and among them, toll-like receptors (TLRs) are well characterized PRRs that can set into play the complex process of inflammation upon recognition of various DAMPs or pathogen-associated molecular pattern molecules (PAMPs) [15,16,17]. This inflammatory response triggers the expression of cytokines, chemokines, interferons and several other associated molecules, and at the same time, it kick starts the adaptive immune system [18,19].

TLRs have a key role in the stimulation of the immune system [19]. Engagement of the TLRs’ ligands transduces intracellular signals by engaging special adaptor proteins that ultimately lead to the activation and nuclear translocation of the transcription factors such as NF-κB and IRFs [19,20]. Both of these transcription factors dictate the consequences of the innate immune response activation. In humans, the TLR family is comprised of 10 members (TLR1–TLR10), while in mice there are twelve (TLR1–TLR9, TLR11–TLR13). In addition to their expression on cell surface membranes, TLRs are found in the endolysosomes, endoplasmic reticulum, lysosomes and endosomes [21]. Structurally these are composed of leucine-rich repeats and an ecto-domain, which arbitrates the PAMPs’ recognition, while a trans-membrane, and Toll/IL-1 receptor (TIR) domain instigates the downstream signaling. The ectodomain exhibits a horseshoe-like structure and through this domain TLRs can ligate their relevant PAMPs or DAMPs [22].

## 2. Myeloid Differentiation Primary-Response Gene 88 (MyD88)

MyD88 is an important downstream protein member of TLR and IL-1 receptors [23]. It was originally discovered by Liebermann and Hoffman in 1990 in M1 myeloblastic leukemia cells where its expression was induced in response to the application of recombinant IL-6 or lung conditioned medium [24]. “MyD” basically stands for myeloid differentiation and “88” denotes the number in the list of induced genes [24]. It is now well established as an adaptor of inflammatory signaling pathways [23]. MyD88 is further associated with IL-1R-associated kinase (IRAK) family kinases through homotypic protein–protein interaction. Stimulation of IRAK at the cellular level initiates various responses, ultimately triggering the nuclear factor-kappa B (NFκB), mitogen-activated protein kinases (MAPK) and activator protein 1 (AP1), highlighting MyD88 as an important mediator of inflammatory pathways [25]. Except TLR3, in all other TLRs sub-stream intracellular signaling is dependent upon MyD88 [19]. Furthermore, advanced studies have revealed that the cellular pathway of the MyD88 signaling is an important mediator of sterile inflammation and it is affected by numerous associated subcellular proteins.

## 3. Role of TLR4-MyD88 in Post-SAH Inflammation

TLR4 is an important member of the TLR family of PRRs responsible for recognizing many pathogenic agonists including various DAMPs and PAMPs [15,16,17]. A few endogenous molecules such as fibrinogen and heme, which are released during SAH are recognized like LPS by the TLR4 [9,26]. When TLR4 is ligated, it triggers a cascade of intracellular signals that initiate the synthesis of pro-inflammatory cytokines, chemokines, and the expression of co-stimulatory molecules [16]. As sterile inflammation is an attribute of SAH, inflammation has gained importance as a target in the discovery of novel drugs against post-SAH inflammation [10]. Among TLRs, TLR4 is unique in that it can transmit downstream signaling via both the myeloid differentiation primary response protein 88 (MyD88) and the TRIF pathways to stimulate and initiate the inflammatory responses [27]. A study showed that during an early stage of SAH, neuronal inflammation and apoptosis was mediated by TLR4/MyD88-dependent and microglial-dependent pathways [28,29]. However, in the late phase of SAH, the neuronal apoptosis was mainly associated with TRIF-dependent and microglial-independent pathways [28,29]. Interestingly, the persistent upregulation of TLR4 at the brain level is linked with long term cognitive dysfunction [30]. This dual pattern of cerebral inflammation is very important because it reveals the importance of TLR4-mediated cerebral effects and proves the importance of this novel therapeutic target for the treatment of the SAH.

**Figure 1:**
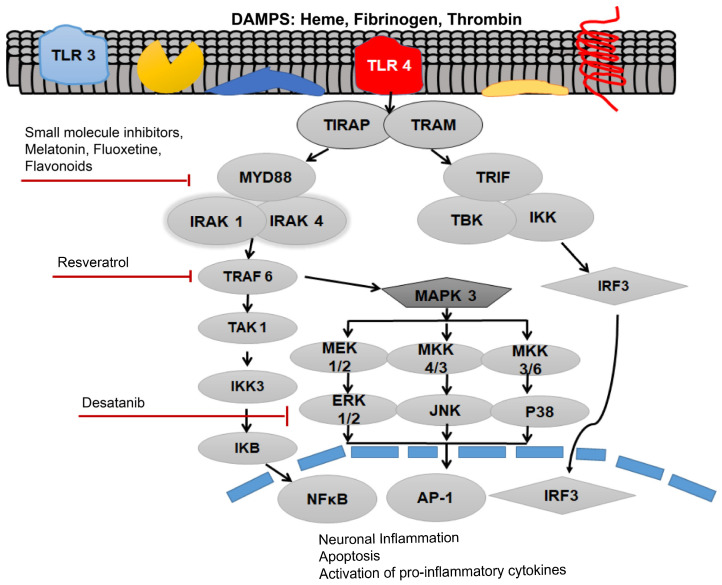
Activation of toll-like receptor 4 (TLR4) via various damage-associated molecular pattern molecules (DAMPs) to initiate downstream signaling of the receptor after subarachnoid hemorrhage (SAH). Myleoid Diffrentiation 88 (MyD88) can be inhibited by the small molecular inhibitor, melatonin, fluoxetine and various flavonoids. TNF Receptor associated factor 6 (TRAF6) can be inhibited by the Resveratrol, whereas Desatanib targets the mitogen-activated protein kinase 3 (MAPK 3) pathway. TLR4 pathway is associated with neuronal inflammation, apoptosis and activation of pro-inflammatory cytokines.

TLR4 is most abundantly expressed on astrocytes, neurons, microglial cells, myeloid cells, and epithelial cells in the central nervous system (CNS) [31]. Recent evidence suggests that TLR4 expression on non-neuronal and non-glial cells also contributes to the brain injury and is implicated in post-SAH complications [31]. Previously, increased levels of soluble isoforms of TLR2 and TLR4 have been observed in the cerebrospinal fluid (CSF) of SAH patients who developed hydrocephalus [32]. A recent investigation showed that in a rodent model of intraventricular haemorrhage, TLR4 mediates the CSF hypersecretion by the choroid plexus epithelium triggered in the cerebral ventricles, resulting in post-hemorrhagic hydrocephalus [33]. Moreover, results have proved that MyD88-dependent TLR4 signaling might play an important role as an inflammatory mediator on the aforementioned cell types in the CNS. Blood-borne DAMPs that bind to the TLR4 act as TLR4 ligands. Initially, DAMPs are identified by a cluster of differentiation 14 (CD-14), either in a soluble or glycosylphosphatidylinositol-anchored form, which then moves to myeloid differentiation factor 2 (MD-2) [31]. Following the ligation of TLR4 agonists, i.e., blood DAMPs, results in TLR4 oligomerization and binding to the TIRAP via TIR–TIR domain interaction as shown in Figure 1. Then, MyD88 associates with this complex. Following this association, this complex recruits further MyD88 molecules, members of the IL-1 receptor-associated family of kinases (IRAKs), and TNF receptor-associated factor 6 (TRAF6), and all of these molecules collectively give rise to the myddosome as shown in Figure 1 [34].

The formation of myddosome directs the downstream activation of transforming growth factor-β-activated kinase 1 (TAK1), which triggers IκB kinase (IKK) and mitogen-activated protein kinase (MAPK) signaling. The phosphorylation of IκB, leads to the activation of the nuclear translocation of nuclear factor kappa-light-chain-enhancer of activated B cells (NF-κB), hence, stimulating the transcription of pro-inflammatory genes including various pro-inflammatory cytokines [29].

Study proves that both MyD88 and the TRIF pathways activate the expression of NF-κB, which is the foremost important transcriptional manager of inflammation-associated genes. The activation of MAPK can initiate the expression of pro-inflammatory cytokines. TNF-α can initiate vasoconstriction and oxidative stress. Furthermore, augmented TNF-α levels, especially in the brain interstitial fluid, further exacerbate cerebral vasospasm [28]. The cytokines are known to worsen the SAH-associated symptoms. IL-1β causes apoptosis, hence it further expedites the cyclooxygenase-2-associated cerebral inflammation. A downstream regulator of the MyD88, ICAM-1 is an important endothelial protein that is usually upregulated, while cerebral inflammation evidence has revealed that it might be a crucial protagonist in microcirculatory dysfunction in SAH [35]. MyD88 further activates the MAPK pathway as shown in Figure 1. MAPK and c-Jun N-terminal kinases (JNKs) signaling causes the downstream activation of activator protein 1 (AP-1), and upregulates the pro-inflammatory cytokine production [27].

MAPKs are directly associated with a lot of important cellular responses and stimuli including mitogens, heat shock, and inflammation. The MyD88-dependent pathway has also been shown to play an extremely important role in cell survival by the activation of MAPKs, such as the signal-regulated kinase (ERK), p38, and c-Jun N-terminal kinase (JNK), in response to which it activates the transcription factor activator protein-1 (AP-1) [36]. Likewise, the MAPK pathway appears to have an important role in the SAH. The MAPK pathway was a key pathway in the regulation of cerebral blood flow in the SAH induced in a rat model [37]. However, both the p38 and JNK were also found to produce neuronal and endothelial cell apoptosis, inflammatory cytokine expression, and facilitate SAH accompanied neuronal injury. In order to understand the role of MAPK pathways in post-SAH injury, recombinant osteopontin (r-OPN) was used in a rodent endovascular perforation SAH model. Pre-SAH intracerebrovascular administration of r-OPN enhanced the expression of an endogenous MAPK inhibitor, MKP-1, through interaction with L-arginyl-glycyl-L-aspartate-dependent integrin receptors and prevented vasospasm after SAH. Inhibition of this MAPK inhibitor by administering MKP-1 siRNA failed to suppress the phosphorylation of MAPKs and led to spastic cerebral arteries in a rat model at 24 h post-SAH [38]. Amazingly, it was shown that the administration of r-OPN associated increase in MAPK inhibitor (MKP-1) not only ameliorated the SAH-associated vasospasm, but also prevented from neurological impairments at 24–72 h post-SAH in a rat model.

## 4. MyD88 as a Therapeutic Target of Post-SAH Inflammation

Recently advanced studies have shown that the inhibition of MyD88-dependent TLR4 signaling is favorable in treating secondary injuries associated with brain hemorrhage. A few potential inhibitors of this pathway are small molecule inhibitors, aptamers, monoclonal antibodies, polyphenols and certain antibiotics.

### 4.1. Progesterone

Progesterone is known to modulate TLR4-MyD88-NF-*κ*B dependent inflammation and reduces the secretion of pro-inflammatory cytokines IL-6 and TNF-α [39]. Progesterone administration has been shown to modulate the inflammation upregulated by TLR4 and downstream signaling pathways during ischemic stroke and traumatic brain injury [40]. Similarly, progesterone administration has also been shown to modulate the TLR4 and downstream pathways-dependent inflammation after SAH in rats [40]. As erstwhile explained, SAH could up-regulate expressions of both TLR4 and NF-*κ*B. Similarly, expression of molecules downstream of TLR4/MyD88 were increased, leading to upregulation of ICAM-1, MCP-1, NF-*κ*B, IL-1β, IL-6 and TNF-*α* after SAH in a rat model [40]. Progesterone administration not only downregulated the post-SAH inflammation by abrogating the expression of these inflammatory molecules in the cerebral tissue, but also improved neurological deficits, brain edema and prevented the disruption of the blood-brain barrier (BBB) [40]. Therefore, progesterone mediated pharmacological modulation of the TLR4-MyD88-NF-*κ*B dependent inflammation after SAH requires further clinical investigation.

### 4.2. Small Molecule Inhibitors

Small molecule inhibitors may be used to pharmacologically target the inhibition of TLR4 and its downstream signaling involving MyD88 to curb the inflammatory response that follows SAH. Interestingly, a small molecule inhibitor of MyD88 dimerization, ST2825, has been shown to alleviate the inflammation during early brain injury after experimental SAH, and it also protects animals against SAH-induced neurological deficits [41]. Intracerebroventricular administration of ST2825 afforded the inhibition of inflammation and apoptosis by modulating the activity of several MyD88 downstream molecules such as TAK1, p38, JNK, NF-κB p65 and IκBα [41]. This study highlights the neuroprotective potential of targeting MyD88 through ST2825 during early brain injury after SAH, however, whether systemic administration of this molecule will provide similar effects to cerebroventricular administration awaits further investigation [41].

### 4.3. Aptamers

A novel approach to counter the inflammation after SAH is the employment of single-stranded DNA aptamers such as ApTLR#1R and ApTLR#4F. Both these aptamers block the TLR4 receptor and act as antagonizing ligands, thus inhibiting the downstream signaling via the TLR4-MyD88 pathway. Both compounds can pass the BBB, and are readily available for absorption, distribution and elimination. Moreover, these agents possess an extremely low toxicity profile. Owing to the pharmacokinetic virtues of these aptamers, rapid systemic administration, theoretically, there is no need for neurosurgical intervention for direct ventricular administration. Novel investigations are required to make these compounds stable because these are prone to degradation by nucleases. Nevertheless, these modifications may compromise the efficacy and toxicity of the aptamers [42]. Both of these aptamers are likely to be TLR4 modulators that are identified for the mitigation of post-SAH inflammation and associated complications.

### 4.4. Polyphenols

Resveratrol, biochanin A and curcumin are polyphenols that are isolated from plants. They can cross the BBB and significantly block the TLR4 signaling by interfering with the oligomerization of TLR-4 [43,44]. Biochanin A evidently reduces the expression levels of TLRs and its downstream signaling proteins like TIRAP, MyD88 and NF-*κ*B pathways and the synthesis of pro-inflammatory cytokines during early brain injury after prechiasmatic blood injection in a model of SAH. Intriguingly, biochanin A administration was associated with reduced neuronal apoptosis, which was linked to improved neurological scores assessed by Garcia Scale scores, and improved spatial learning and cognitive memory assessed by modified water maze tests after SAH [45].

Resveratrol (3,4′,5-trihydroxystilbene) has been suggested to reduce inflammation across various neurological disorders [46]. A previous study investigated the beneficial effects of resveratrol in a SAH model, showing inhibition of the translocation of NF-*κ*B, which was associated with reduced pro-inflammatory cytokines expression, reduced expression of MMP-9 and increased expression of junctional proteins [47]. All of these effects led to reduced mortality, improved neurological scores, reduced BBB disruption and consequent edema [47]. A later detailed experimental SAH study showed a significant reduction in acute inflammation upon resveratrol administration after SAH [46]. Resveratrol administration inhibited the expression of HMGB1, TLR-4, MyD88, NF-*κ*B, which was associated with inhibition of microglial activation, reduced neuronal apoptosis, brain edema, BBB disruption and improved neurological scores [46]. Furthermore, resveratrol has been suggested to diminish adenosine diphosphate-induced platelet aggregation [31]. Consequently, inhibition of the platelet accumulation at the site of the lesion may be an additional mechanism, whereby resveratrol provides neuroprotection. Resveratrol effectively reduces the release of the pro-inflammatory cytokines [46,47]. Polyphenols are known to diminish the translocation of NF-κB into the nucleus, potentially inhibiting the expression of pro-inflammatory genes [46]. Very interestingly, a study investigating the role of curcumin in a SAH model has shown that curcumin alleviates inflammation by inhibiting the TLR-4/MyD88/NF-*κ*B inflammatory axis and promotes the polarization of microglia towards a neuroprotective phenotype—the M2 (alternatively activated) microglia [44]. Further clinical investigations are required to determine the therapeutic effectiveness of these compounds.

### 4.5. Melatonin

Melatonin, a pleiotropic hormone produced by the pineal and extrapineal tissues regulates the circadian rhythms. However, this tryptophan derivative has important anti-inflammatory and immune modulatory properties [48,49,50]. In the SAH-induced rats by prechiasmatic cisternal injection of the autologous blood, treatment with melatonin has been shown to significantly reduce the expression of HMGB1, TLR4 and downstream proteins like NF-κB, MyD88, relevant pro-inflammatory cytokines such as IL-1β, TNF-α, IL-6, and inducible nitric oxide synthase. Importantly, the administration of melatonin after SAH significantly improved the memory and spatial learning as assessed by a water maze along with reduced neuronal apoptosis. This is consistent with the notion that melatonin displays neuro-protection not only via the anti-oxidant pathway, but also via anti-inflammatory signaling [51]. Clinical investigations aimed at melatonin-led improvements in post-SAH complications and clinical outcome stemming from reduced inflammation after SAH are required.

### 4.6. Monoclonal Antibodies

The activation of the MyD88 pathway enhances the expression of the pro-inflammatory cytokines. Direct cytokine inhibitors like monoclonal antibodies can be used for the significant reduction of SAH-associated inflammation. It was observed that the monoclonal antibody mediated targeting of TNF-α could be useful therapeutic target for SAH. Infliximab and canakinumab target the TNF-α and IL-1β, respectively [31]. The disadvantage of these antibodies are that they are mostly incapable of moving across the BBB and it requires extra measures to access the cerebral region. Nevertheless, in ischemic stroke, it was reported that infliximab can cross the BBB in the diseased condition. This is also true in case of SAH as the BBB is also disrupted and amenable to the therapeutic potential of these antibodies [8,31,52]. However, preliminary data suggests that the ICU stay of many SAH patients is usually prolonged because patients are often intubated and immobile, which greatly increases the chances of infection in the patients. Owing to the use of immunosuppressants, such patients are always prone to infections [31]. Therefore, cautious investigations regarding the clinical use of these immunosuppressing monoclonal antibodies are required without increasing the frequency of infections.

### 4.7. Pentoxifylline

Pentoxifylline is known as a nonselective phosphodiesterase inhibitor. Several studies have shown that pentoxifylline has anti-inflammatory and neuroprotective properties in a number of brain injury animal models. Pentoxifylline has been reported to inhibit the TLR4 and downstream signaling molecules such as MyD88 and NF-κB. Moreover, it possesses the potential to significantly reduce the pro-inflammatory cytokines, and reduce neural cell death and BBB permeability, highlighting another important clinically used drug that needs to be investigated in clinical trials in SAH patients [53].

### 4.8. Astaxanthin

Neuroinflammation is a hallmark of SAH and other cerebral injuries. Astaxanthin is a dietary carotenoid that possesses anti-inflammatory properties. Post-SAH treatment with astaxanthin can considerably down-regulate the MyD88 and NF-κB activity. Furthermore, inhibition of TLRs can improve cerebral edema, blood–brain barrier disruption, neurological dysfunction, and neuronal degeneration. As astaxanthin has anti-inflammatory potential and it retains neuro-protective potential against SAH, further investigations in clinical settings are needed [54].

### 4.9. Fluoxetine

Fluoxetine is a well-known antidepressant drug belonging to the selective serotonin reuptake inhibitors and its neuroprotective effects have been described in various neurological disorders [55]. Interestingly, fluoxetine administration in an endovascular perforation model of SAH has shown anti-inflammatory effects through the down regulation of TLR-4, MyD88, NF-κB, pro-inflammatory cytokines expression, reduced activation of microglia and infiltration of neutrophils, and improved neurobehavioral outcomes due to reduction in neuronal loss, brain edema and protection of junctional proteins [55]. Interestingly, fluoxetine also offers neuroprotection during early brain injury after SAH due to the inhibition of inflammasome formation and consequent necrotic death [55].

## 5. Discussion

A large body of evidence highlights the role of TLR4-MyD88 downstream signaling after SAH [28,29,44,45,46,51,53,54,55,56]. Neuroinflammation is a known consequence of SAH, and MyD88 may be a vital pharmacological target for the amelioration of post-SAH inflammation. It is well-recognized that the TLR4 signaling pathway has an important role in altering normal neuronal functions [57]. The hallmark of the TLR4-MyD88 signaling pathways are the development of neuronal apoptosis, BBB disruption, and cerebral vasospasm. However, various studies have suggested that both NF-κB and MAPK pathways underlying MyD88 are implicated in the development of cerebral vasospasm. Increasing evidence has shown that TLR4 signaling plays an important role in SAH-induced brain injuries [32]. Moreover, a basic scientific study with genetic mice models revealed that the MyD88 signaling pathway in endothelial cells of the cerebral microvasculature, but not in the microglial cells, is a main and very versatile mediator of systemic immune activation. These fine mechanisms taking place at the level of the blood-brain barrier seem to be able to inhibit cells of the innate immune system and diseases that have immune etiologies such as SAH [58].

Several small molecule inhibitors that abrogate TLR-MyD88-NF-κB mediated inflammation represent potential pharmacological therapies for SAH patients. For instance, a known drug, Dasatinib is a small molecule that has been shown to suppress microglial activation, neutrophil infiltration, and pro-inflammatory cytokine levels, and also, it can cross the BBB. Moreover, in order to decrease inflammation, Dasatinib inactivates TLR4 and associated downstream effectors like protein kinase B (AKT) and extracellular receptor kinase (ERK) [59]. T6167923 and ST2825 are also small molecules that are known as specific inhibitors of TLR4 signaling. ST2825, as discussed above, has been shown to protect against inflammation after SAH. More specifically, these bind with the BB-loop of the TIR domain on MyD88, hence, thwarting MyD88 homo-dimerization. This is associated with the abrogation of the signaling involving MyD88, TIRAP and TLR4, and therefore, myddosome formation [60,61].

The naturally occurring de-hydroabietic acid 40 and synthetic 5z-7-oxozeanenol l41 have been shown to suppress MyD88 inflammatory signaling via TAK1 inhibition, whereas BAY 11-708242 targets IKK, subsequently leading to the inhibition of NF-κB activation. De-hydroabietic acid can cross the BBB effectively, however, other mentioned compounds cannot do the same in normal conditions [62]. Although these molecules have not been investigated and studied in animal or human models of SAH, these molecules have shown MyD88-dependent signal depletion and further research is needed to explore the pharmacological potential of these molecules.

Other molecules such as LPS-RS and IAXO-102 possess the potential to impede cerebral vasospasm in SAH induced in mice. LPS-RS interact with MD-2-TLR4 and IAXO-102 deters the action of CD-14, while MD-2.33 LPS-RS has also been proved to abate TLR4, JNK, and p38 activation, effectively mitigating their effects, i.e., vasoconstrictive effects. Both compounds have proved to be effective in diminishing SAH associated inflammation. Their use requires intracerebroventricular (ICV) administration because they are not permeable to BBB [63].

Amazingly, levofloxacin and ciprofloxacin are capable of exerting inhibitory effects on TLR4-mediated inflammation by acting on the TLR4 receptors. These drugs impart anti-inflammatory effects by binding with the hydrophobic regions of the receptor, thus affecting the dimerization of TLR4 that is needed for activation of the particular cellular pathway [64]. Hence, these drugs can inhibit the downstream signaling of the TLR including the MyD88 pathway. Additionally, both these fluroquinolones are clinically used for the treatment of bacterial meningitis, while in diseased states, fluroquinolones are capable of crossing the BBB [65]. Further research is required to explore their anti-inflammatory effects and possible use in the SAH. Several compounds that are known to inhibit the neuroinflammation in other diseases can also be explored for the possible reduction of inflammation associated with the SAH. Advanced computational in-silico studies can also be employed in this regard.

Low doses of heparin and self-assembling heparin nanoparticles are currently considered as good options for the treatment of SAH. The infusion of low dose heparin over 24 h has been used in the post-SAH management of patients. Several studies have indicated the beneficial role of heparin. By using low dose heparin and its nanoparticles, it was observed that cognitive outcomes were better and the occurrence of vasospasm was reduced and cerebral ischemia was deferred for a brief period [66]. Nevertheless, advanced studies have proved that heparin has a diminutive effect on vasospastic damage, especially SAH-associated whereas it may still be effective in lowering inflammatory-related injuries that are related to SAH [67]. The underlying anti-inflammatory properties of heparin may stem from its ability to ameliorate both MAPK-p38 and NF-κB inflammatory pathways downstream of the MyD88 adaptor [68].

It has been suggested that heparin, or heparin-derived drugs, could decrease neuro-inflammatory-related injury in SAH. However, the current data is preliminary and clinical research is required to establish the therapeutic use of heparin in SAH. A recently completed study in a blinded multi-center Phase II clinical trial verified the utilization of heparin administration in aneurysmal SAH (ClinicalTrials.gov Identifier: NCT02501434).

Still, extensive research is required to completely understand the pathogenic role of TLR4 in SAH in order to further establish the underlying mechanism through which several MyD88 antagonists may ameliorate the disease. Hence, the information compiled here proposes that the inhibition of the MyD88 signaling cascade could be a promising therapeutic option for managing injuries secondary to brain hemorrhage. Therefore, all the inhibitory molecules that have been discussed require further, extensive clinical evaluation [33].

A better understanding of the TLR4-MyD88 signaling in SAH will expedite the development of novel therapeutic options. It was found that while TLR4 signaling pathways are detrimental in both the early and late phase of SAH, however, prolonged treatment with TLR4 inhibitors might be toxic and halt regeneration. As discussed before, endogenous ligands activate the TLR4 pathway, but it is still undetermined which of these ligands are the most significant and potent and how these ligands activate several TLR4 signaling pathways. There is also a need to determine the nature and structural conformation of TLR4 and associated signaling pathways, either akin to all cells within a human being or in any other species [32]. Furthermore, the possible targets for the treatment of SAH in the TLR4 signaling pathway include the extracellular binding proteins (MD-2 and CD14), downstream pathways, i.e., adaptor proteins (MyD88 and TRIF), MAPKs and transcription factors (NF-κB, and AP-1), but the most effective target for treatment is still to be determined [69]. In order to solve these mysteries, further studies should be conducted. In pre-clinical studies, novel treatment possibilities affecting TLR4 have proved to be extremely effective for preventing SAH and its associated effects. Of note, current research indicates that exploiting stem cell technologies presents a scientific rationale, and with the recent penetration of clinical applications of neural stem cell transplantation, this translates into a feasible option to treat SAH and post-treatment complications [70]. As miss-regulation of microglial MyD88 signaling was shown to misbalance neural stem cell maintenance in the hippocampus [71], any neuro-repair or general cell-based endeavors to treatment of SAH should carefully monitor, and ideally, control MyD88 activity in the cell therapeutics and/or host tissue, respectively.

## 6. Conclusions

The MyD88 gene expresses the MyD88 protein that plays a significant role in the progression of inflammation upregulated after SAH. MyD88 is an adaptor protein that worsens SAH symptoms. We also explain the role of MyD88 in the disease progression. Although there are multiple hurdles to develop successful pharmacological agents to treat the post-SAH inflammation, a few agents directly inhibit the MyD88 whereas a few agents further inhibit the downstream regulators of this pathway. However, in this review, we have discussed several extremely promising pharmacological agents that can be used therapeutically in such conditions. Further clinical and toxicological studies are required to establish their role in SAH. A brief summary of some of the interventions modulating the TLR4-MyD88 pathway are represented in Table 1.

## 7. Methodology

A systematic search for potential articles to be included in this review was conducted using Pubmed. The combination of the following terms was searched as MeSH terms or as words in any field to build the initial library; MyD88, subarachnoid hemorrhage, MyD88 protein. This search strategy yielded 15 articles conforming to the given criteria. Further, the bibliographies of these articles were explored to include any additional articles.

## Figures and Tables

**Table 1:** MyD88-dependent TLR4 signaling inhibitors.

Pharmacological Agent	Drug Target	Dose	Animal Model	Mechanism	Reference
ApTLR#1R	TLR4	0.1 and 1 nmol i.p. 6 h,12 h and 24 h	CCA and MCA ligation of Mice	Bind to TLR4, avoiding the ligand Binding	[42]
ApTLR#4F	TLR4	0.1 and 1 nmol i.p. 6 h,12 h and 24 h	CCA and MCA ligation of Mice	Bind to TLR4, avoiding the ligand Binding	[42]
Astaxanthin	MyD88, NFKB	0.1 and 0.2 mmol i.p. 12 h and 24 h	Prechiasmatic cistern SAH	Inhibit the NFKB activity	[46]
Ciprofloxacin & Levofloxacin	MD-2	100, 200, 500 mg/kg i.v. 24 h	Prechiasmatic cistern SAH	Blocks the dimerization of TLR4 by interacting with hydrophobic region of MD-2, inhibiting the MD-2 TLR4 interaction	[53,65]
Curcumin	MyD88, TLR4	200 mg/kg i.p. 15 min	Prechiasmatic cistern SAH	Targets TLR4, preventing TLR4 dimerization upon ligand binding	[44]
Dasatinib	TLR4, MyD 88, ERK-AKT	20 mg/kg i.p for 4 days 20 mg/kg orally for 2 weeks	LPS induced neuro-inflammation	Mechanism of action is unknown but suggested to bind and inhibit TLR4, pERK, and pAKT	[59]
Fluoxetine	*TLR4*	10 mg/kg i.v 6 h and 12 h	CCA ligation of mice	Unknown mechanism	[55]
Melatonin	TLR4/Myd88	0.5 or 1 mg/kg i.p.	LPS induced neuro-inflammation	Inhibit the TLR 4 and MyD88 cascade	[51]
Pentoxyfyline	TLR4	60 mg/kg i.p. 6 h and 24 h	Prechiasmatic cistern SAH	Inhibits the TLR4 signaling	[53]
Progesterone	MyD88	16 mg/kg 1,6 and 24 h	Prechiasmatic cistern SAH	Inhibit the MyD88	[40]
Resveratrol	TLR4/TRAF6	60 mg/kg i.p. 6 h and 24 h	Prechiasmatic cistern SAH	Interferes with TLR4 dimerization and mitigates TRAF6 ubiquitination and activation of downstream mediators	[43]
ST2825	MYD88	10, 30, 50 mmol i.p.	-	Mimics and directly binds to the TIR domain on MyD88, preventing MyD88 homodimerization and further signaling	[62]
T6167923	MyD88	10, 30, 50 mmol i.p.	-	Mimics and directly binds to the TIR domain on MyD88, preventing MyD88	[63]

CCA: Common carotid artery, MCA: Middle cerebral artery, i.p.: intraperitoneal, i.v.: intravascular.

## Data Availability

Not applicable.
